# Combined Pulmonary Fibrosis and Emphysema Syndrome: A New Phenotype within the Spectrum of Smoking-Related Interstitial Lung Disease

**DOI:** 10.1155/2012/867870

**Published:** 2012-02-09

**Authors:** Karina Portillo, Josep Morera

**Affiliations:** Pulmonary Department, Hospital Germans Trias i Pujol, Carretera de Canyet s/n, 08916 Badalona, Barcelona, Spain

## Abstract

Combined pulmonary fibrosis and emphysema (CPFE) is a recently defined syndrome, in which centrilobular and/or paraseptal emphysemas in upper lung zones coexist with pulmonary fibrosis in lower lobes in individuals. These patients have a characteristic lung function profile, with unexpected subnormal dynamic and static lung volumes, contrasting with a significant reduction of carbon monoxide transfer (DL_co_) and exercise hypoxemia. Pulmonary hypertension is highly prevalent in CPFE and is the leading determinant of death. Tobacco smoking has been proposed as the main factor in its etiology, though the pathophysiology and its natural history remain to be determined. High-resolution computed axial tomography is the mandatory tool to confirm the diagnosis. Currently, there is no consensus about its treatment since those published to date on this issue are limited to well-characterised series of cases; hence, a better understanding of this entity may help in the development of future therapeutic approaches.

## 1. Introduction

Pulmonary emphysema and idiopathic interstitial lung diseases (ILDs) including idiopathic pulmonary fibrosis (IPF) are entities defined by different clinical, functional, radiological, and pathological criteria [1]. IPF is the most common ILD, occurring primarily in older adults, and associated with the histopathologic and/or radiologic pattern of usual interstitial pneumonia (UIP) [2]. Emphysema is defined as an enlargement of the air spaces distal to the terminal bronchioles due to the destruction of the tissues forming their walls [3]. Emphysema can cause an obstructive pattern due to the different structural changes occurring in the lung [3–5].

Traditionally regarded as separate disease states, combination of both processes was described over 30 years ago by Auerbach et al. [6] in a pathological study of 1824 autopsy lungs. Even then, authors suggested that smoking could be responsible for the coexistence of these processes, basing on studies involving animal models exposed to tobacco smoke [7, 8]. In early nineties, by means of high-resolution computed axial tomography (HRCT), Wiggins et al. [9] reported a correlation between functional and radiologic findings in 8 subjects with history of smoking who had severe dyspnea, but whose pulmonary functional tests revealed no signs of obstruction, normal static lung volumes, and depressed DL_CO_. Imaging study showed that these patients had areas of upper-lobe predominant emphysema and lesions compatible with fibrosis in both lung bases. In 2005, Cottin et al. characterized this condition for the first time as a well-defined syndrome termed “combined pulmonary fibrosis and emphysema” (CPFE) [1]. Since then, there is increasing interest in this new entity, and several series, most with a retrospective methodology, have been published in the literature [10–25].

In this paper, we will review the particular features of CFPE and also discuss some of the most recent evidence published.

## 2. Epidemiology

The prevalence of CPFE is not known although it has been estimated to represent between 5% and 10% of cases of diffuse interstitial lung disease [26]. Most of the cohorts studied have been men and generally present in people over 65 years of age who are active smokers or heavy ex-smokers (over 40 pack years). Exposure to agricultural compounds is another epidemiological data collected by some series [1, 20, 27].

Recently, Cottin et al. [24] described the association of CFPE with connective tissue diseases (CTDs). Several differences were observed between this cohort and population described in other series with CPFE. Although the majority of patients with associated CTD had a similar smoking history and similar function profile compared with “classic CPFE,” the populations studied were significantly younger, were more likely to be women, and tended to have less severe outcomes. The predominant CTDs were rheumatoid arthritis followed by systemic sclerosis and mixed connective tissue disease. These observations should be explored in future studies in order to analyse whether underlying inflammatory disease also predisposes to the development of CFPE with different presentation.

Limited but interesting epidemiological data are available about the relationship between CPFE and lung cancer. Usui et al. [23] reported a high prevalence of CPFE in 1143 individuals with lung cancer. Specifically, CPFE was identified in 101 cases (8.9%). These patients had a history of heavier smoking and poorer prognosis than the ones without CPFE. Similarly, Kitaguchi et al. [20] retrospectively reviewed the records of 47 patients with CPFE and found that 22 of those patients (46.8%) had lung cancer. Given that these findings have been described in asiatic populations is unknown to date if an ethnic or genetic component enhanced by smoking exposure is also involved.

## 3. Clinical Characteristics

Exertional dyspnea (functional class III or IV of the New York Heart Association) is the most common symptom among patients. Physical examination usually reveals bibasilar inspiratory crackles and finger clubbing. Although less common, other signs and symptoms reported are cough, wheezing, perioral cyanosis, and asthenia [1, 13, 21, 26].

Pulmonary hypertension is a common and important complication in the natural history of this syndrome, as it is associated with a worse clinical course and lower survival rates [1, 14, 26, 28]. Additionally, several authors have reported a significant prevalence of cardiovascular disturbances in these patients such a atherosclerotic artery disease (coronary and peripheral) and left ventricular diastolic dysfunction on echocardiography [22].

## 4. Pathogenesis

Despite the lack of knowledge regarding the physiopathologic substrate of CPFE, it can be supposed that it entails a complex, overlapping process involving different types of cells, shared pathways, and mediators with an inflammatory and/or fibrogenic capacity. This process finally leads to the destruction of the lung parenchyma and the aberrant remodelling characteristic of lung fibrosis. Furthermore, these cell mediators interact with environmental factors which could act as modifiers of the disease in the presence of a permissive genotype. The role of tobacco smoking is suggested by its demonstrated causative agent in both emphysema and IPF [24]. Experimental studies in animal models are providing information on the involvement of some inflammatory mediators in the development of CPFE. The identification of molecular and genetic alterations involved in the etiology of this syndrome through other methods (i.e., surgical lung biopsies, explants from lobectomies) is needed, since it also will provide a more effective approach for early diagnosis and identifying molecular targets for future therapies.

### 4.1. Etiology

Cigarette smoking has been suggested as the main etiologic factor, as a history of smoking is a constant factor in all the cohorts reported [29]. Tobacco smoke, a complex mix of over 4000 chemical substances, has been associated with a wide range of diseases, and emphysema is the lung disorder most commonly attributed to smoking [30]. Kaolinite or aluminium silicate is an inorganic industrial material found in tobacco smoke and which has been isolated in alveolar macrophages of smoker patients with lung emphysema and respiratory bronchiolitis with diffuse interstitial lung disease (RB/DILD) [31]. It has been hypothesized that macrophage accumulation after chronic inhalation of this mineral triggers the series of physiopathological events that, in the end, lead to respiratory bronchiolitis and emphysema [32].

Although the etiology of IPF is unknown, it is thought to be a consequence of the interaction of environmental factors in individuals with a genetic predisposition, with increasing recognition of its association with smoking [33, 34]. In this context, Katzenstein et al. [35] reported an unexpectedly high frequency of fibrosis in pieces of lung tissue removed from smokers who were candidates for tumor surgery. These patients showed no clinical evidence of interstitial lung disease. It should be pointed out that smoking has also been associated with a restrictive spirometric pattern in many epidemiological studies [36], which supports the hypothesis that smoking can produce different types of effects on the lung parenchyma, as evidenced in different phenotypic expressions.

The association between CPFE and lung cancer may reflect the susceptibility to chronic smoking-induced inflammation, as shown in other studies on the relationship between COPD and IPF [23, 37, 38]. In addition, the high prevalence of vasculopathy and pulmonary hypertension described in CPFE can also support the role of smoking habit as pathogenetic factor, since it is a recognized risk factor in the development of blood vessel disorders. The exposure of endothelial cells of the pulmonary arteries to tobacco smoke extract has been shown to cause an irreversible inhibition of endothelial nitric oxide synthase (eNOS) activity due to a reduction in its protein content and messenger RNA. This inhibition of eNOS activity due to tobacco smoke may explain the decreased expression in pulmonary arteries seen in smokers and may predispose patients to greater changes in the pulmonary vessels [39].

### 4.2. Oxidative Stress

Increases in oxidative and nitrosative stress have been postulated as mechanisms potentially involved in the development of CPFE; they increase inflammatory-cell (leukocyte) activation, which contributes to local and systemic increases in oxidant levels. The production of an excess of oxidants that are not neutralized by the body's antioxidant systems causes structural changes in epithelial, vascular, and connective tissues [35].

### 4.3. Metalloproteinases

Metalloproteinases (MMPs) constitute a family of enzymes produced by alveolar epithelial cells, macrophages, and neutrophils, which participate in emphysema development due to their significant proteolytic activity and collagen-degrading ability. The expression of MMP activity is modulated by several cytokines, including interleukin 13 (IL 13), which, in experimental models, is able to produce enlarged airway spaces, fibrosis, and inflammatory remodelling of the airways [40]. A recent study in a subgroup of CPFE patients reported an increase in the expression of some MMPs in the areas affected by a combination of emphysema and UIP, suggesting that these proteins also have a role in extracellular matrix deposition and anomalous tissue remodelling— a characteristic hallmark of the process of lung fibrosis [30].

### 4.4. Caveolin-1

Caveolae are invaginations of the cell membrane in various types of cells. They are rich in proteins, lipids, and cholesterol and are formed and maintained by proteins called caveolins and caveolin-1 being the most studied. This protein is a potent immunomodulator, and it has been ascribed several functions, including signal transduction, the mediation of cell apoptosis, intracellular calcium and eNOS regulation, and the suppression of tumours [41, 42]. Lung tissue expresses high levels of caveolins, and dysfunctions have been linked to pulmonary hypertension associated with COPD and emphysema, interstitial fibrosis, and lung cancer. As a result, caveolin-1 may be another important mediator in the etiology of CPFE, and it undoubtedly deserves to be the subject of further investigations [43].

### 4.5. Genetic Factors

Mutations in the surfactant protein C (SFTPC) gene have been mostly associated with interstitial lung disease [44]. Cottin et al. [45] recently reported a dominant mutation in the SFTPC gene in a young nonsmoking female with CPFE and in her infant with interstitial lung disease. The pathophysiology of SP-C-associated disease involves the dysfunction of surfactant homeostasis, causing injury or death of alveolar epithelial type II cells and myofibroblast proliferation. A process of genetically mediated alveolar injury may conceivably contribute to emphysema in addition to inflammation and fibrosis, and thus to the CPFE phenotype. This first paper supports the hypothesis that patients with CPFE syndrome may have an underlying genetic predisposition.

### 4.6. Animal Models

Three studies using animal models have managed to reproduce the histopathological elements of CPFE. The first, performed by Hoyle et al. [46], showed that the overexpression of platelet-derived growth factor (PDGF) led to the enlargement of airway spaces, and inflammation and fibrosis in the lungs of transgenic mice. PDGF is a growth factor and has a pleiotropic effect on several lines of cells and is involved in the pathogenesis of both IPF and emphysema. This dual action may be due to its mitogenic activity on fibroblasts, its interaction with different inflammatory markers, and its probable capacity to induce a protease/antiprotease imbalance in the extracellular matrix, which would trigger the development of emphysema.

Overexpression of other mediators in the lungs, such as tumor necrosis factor-alpha (TNF-*α*) and transforming growth factor-beta (TGF-*β*), has also generated this complex phenotype of fibrosis and emphysema in research animals [47, 48]. TNF-*α* is a multifunctional cytokine with some apparent profibrotic activity and is considered an important mediator of various pulmonary and systemic symptoms in diverse respiratory diseases [49]. Smoking causes this cytokine to be released into the lungs, both in humans and animal models, and high levels have been found in the sputum and peripheral blood of COPD patients [50].

TGF-*β* is one of the most potent profibrogenic mediators known to play a role in the pathogenesis of IPF. The TGF-*β*1 isoform induces the differentiation of fibroblasts into myofibroblasts, the transition of epithelial cells into fibroblasts, and the synthesis of molecules of the extracellular matrix, as well as promoting the apoptosis of alveolar epithelial cells [49]. Its role in the development of emphysema is less clear although an increase in its expression in the bronchial epithelium and in the macrophages of the small airways has been observed in COPD patients [51]. In summary, TGF-*β* is an important mediator during the transition from the immune inflammatory response to the tissue remodelling process [52, 53].

## 5. Diagnosis

### 5.1. Imaging Studies

**Figure 1:**
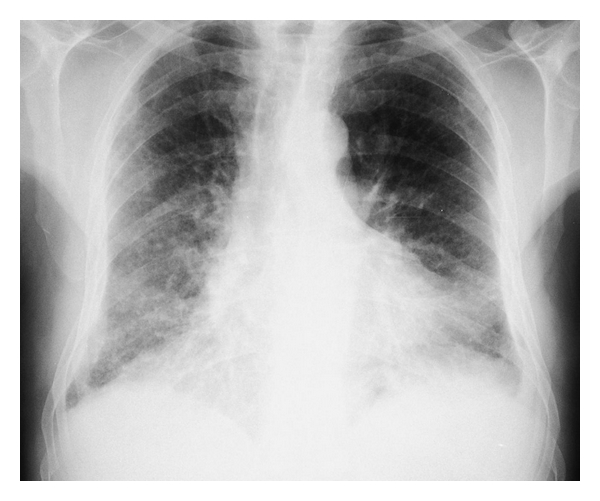
Chest X-ray of a patient diagnosed with combined pulmonary fibrosis and emphysema demonstrating bilateral interstitial pattern, predominantly right sided, with reticulonodular infiltrates in the lung bases and subpleural region, and a reduction of lung density in upper lung fields, mainly on the left.

**Figure 2:**
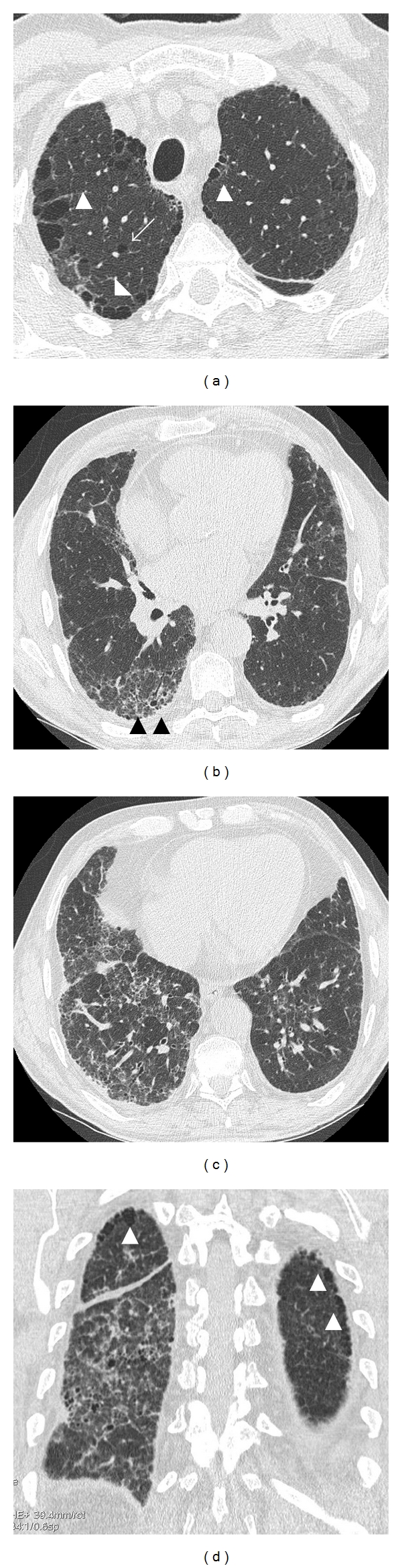
High-resolution computerized tomography (HRCT) of the same patient. (a) Presence of paraseptal emphysema and subpleural bullae (white arrowheads) and centrilobular emphysema (arrows) in both upper lobes. (b) Reticular interstitial disease with intralobular thickening and images of subpleural honeycombing and traction bronchiectasis (black arrowheads), (c) Reticular interstitial disease in middle and right lower lobes, with interlobular septal thickening, subpleural honeycombing, and traction bronchiectasis. (d) Coronal reconstruction in the posterior regions of both lungs: bilateral paraseptal emphysema (white arrowheads) and reticular interstitial disease and honeycombing in right lower lobe.

A simple chest X-ray reveals an interstitial pattern or reticulonodular infiltrates in both lung bases and the subpleural region, and hyperlucency in the apices with thinning of pulmonary vessels and a reduction in their number. (Figure 1). However, the radiologic findings for this entity may go unnoticed in a chest X-ray, so HRCT scanning is the most appropriate technique for confirming diagnosis. (Figure 2) Cottin et al. [1] described the following radiologic criteria to determine the CFPE: firstly, the presence of emphysema on HRCT, defined as well-demarcated areas of decreased attenuation in comparison with contiguous normal lung and marginated by a very thin (<1 mm) wall or no wall, and/or multiple bullae (>1 cm) with upper zone predominance, and secondly, the presence of diffuse parenchymal lung disease with significant pulmonary fibrosis on HRCT, defined as reticular opacities with peripheral and basal predominance, honeycombing, architectural distortion, and/or traction bronchiectasis or bronchiolectasis; focal ground-glass opacities and/or areas of alveolar condensation may be associated but should not be prominent. The emphysematous lesions should be graded as a percentage of affected lung, which has to be more than 10% [14].

Centrilobular emphysema and/or bullous emphysema are present in the upper zones in most of published cohorts, and their association with paraseptal emphysema (low-attenuation areas in subpleural zone) has been described in 90% of cases. Hence, some authors suggest that it is a typical feature of CPFE [1, 26, 54, 55].

 In some cases, distinguishing images of emphysema from those of fibrosis is a complex task as a transition area can be observed between both regions, making it difficult to interpret them correctly [55]. For instance, emphysematous changes or cysts surrounding ground-glass opacity may be mistaken for honeycomb cysts [56]. Brillet and colleagues [55] identified three HRCT patterns in 61 patients with CFPE: (1) progressive transition (*n* = 23,38%) with diffuse emphysema (centrilobular and/or bullous) and zone of transition between bullae and honeycombing; (2) paraseptal emphysema (*n* = 13, 21%) with predominant subpleural bullae of enlarging size at the bases; (3) separate processes (*n* = 14, 23%) with independent areas of fibrosis and emphysema. Eleven patients (18%) could not be classified.

The wide variety of radiological signs present in the HRCT scans correlates closely with histopathological data. UIP is the most common pattern, but lesions have also been reported which are compatible with nonusual interstitial pneumonia, tobacco-related ILD, or even other unclassifiable fibrotic lung disease [15]. Given this pathologic heterogeneity, some authors advocate for the nonrequirement of a specific histopathologic pattern as a diagnostic criterion of CPFE [22].

### 5.2. Lung Function and Gas Exchange

The coexistence of emphysema and fibrosis leads to a characteristic functional profile which is in direct contrast to the degree of dyspnea suffered by these patients. Forced vital capacity (FVC), forced expiratory volume in the first second (FEV_1_), and total lung capacity (TLC) are usually within unexpected normal ranges or only slightly abnormal, unlike DL_CO_, which is significantly reduced. Hypoxemia is a common finding; it is generally moderate at rest and gets worse during exercise [1, 17, 26]. Hyperinflation and an increase in pulmonary compliance due to the loss of elasticity in the areas with emphysema probably compensate for the loss of volume caused by fibrosis [16–18]. In contrast, the overlapping of both pathologies could exert a negative synergic effect on gas exchange, resulting in a severe decrease in DL_CO_. This particular functional pattern leads to at least 2 important clinical repercussions: firstly, the presence of normal lung volumes does not exclude the diagnosis of pulmonary fibrosis, and secondly, neither FVC nor TLC can be used as parameters to monitor the disease, as they do not reflect the degree of functional impairment. In this instance, DL_CO_ is the variable which best correlates with the degree of parenchymal destruction. However, a low DL_CO_ can also reflect disorders in the pulmonary vascular bed, specifically pulmonary hypertension (PH), as it is a highly prevalent condition in this entity.

### 5.3. Pulmonary Hypertension

PH is a common complication during the clinical course of CPFE and is the main condition that influents its evolution and prognosis [1, 14, 26, 28]. The prevalence reported in these patients varies between 47% and 90% and is much higher than in COPD or IPF alone [57]. In most published series, the diagnosis of PH was established by a transthoracic echocardiogram. PH was defined by an estimated systolic pressure in the pulmonary artery (eSPAP) ≥ 45 mm Hg. In the study by Cottin et al. [1], the presence of PH was an independent predictor of mortality, with a hazard ratio of 4.03 (*P* = .03). The 5-year probability of survival was 25% in patients with PH diagnosed by echocardiogram compared with 75% in those with no signs of PH at the time of diagnosis. The mean survival time in this series was 6.1 years, dropping to 3.9 years in those patients with associated PH.

Mejía et al. [14] recently published a study in which different clinical, functional, and prognostic variables were compared in a group of patients with CPFE and another with IPF and with no evidence of emphysema. Using a logistic regression model, they showed that, together with the FVC, an eSPAP ≥ 75 mm Hg was one of the main variables determining survival. In these patients, the extent of the emphysema established by HRCT showed a positive correlation with eSPAP.

The significant effect of pulmonary hemodynamics on mortality in this syndrome was shown in another study performed by Cottin and colleagues [28], involving 40 patients with CPFE who had PH confirmed by right heart catheterization. The factors associated with a worse prognosis were increased pulmonary vascular resistance, reduced cardiac index, a high heart rate, and low DL_CO_. In this study, the diagnosis of PH was established at a mean of 16 months after the initial diagnosis of CPFE, and the estimated survival rate at 1 year in this cohort was 60%.

Therefore, routine screening of these patients for PH with Doppler echocardiography would be justified given its high prevalence and its important role in the prognosis. It should be noted that most recent updated clinical classification of PH includes CPFE in the group 3 (owing to lung diseases and/or hypoxia) under the term “Other pulmonary diseases with mixed restrictive and obstructive pattern” [58].

The presence of emphysema can make difficult interpretation of echocardiographic studies, so some authors propose assessing alternative diagnostic techniques such as magnetic resonance imaging (MRI). MRI flow mapping shows good correlations with hemodynamic parameters [59, 60]. However, general access and use of this technique in this field is still very limited.

## 6. Treatment

The therapeutic options for CPFE are limited. Treatment with systemic corticosteroids and immunomodulator therapy, similar to that for treating IPF, has been used, but without beneficial results in the published series. Smoking cessation is a sensible measure that probably halts the progression of emphysema lesions. Oxygen therapy is appropriate for management of hypoxemia.The possibility of using the specific therapy approved for treating pulmonary arterial hypertension (endothelin-1 receptor antagonists, prostanoids, or phosphodiesterase type 5 inhibitors), as has been tested in COPD or IPF, has been considered by some authors. However, no studies have been published to date on this issue. It is important to point out that the presence of emphysema and abnormal changes in pulmonary vascular bed in these patients may be associated with an imbalance in the ventilation/perfusion ratio (V/Q), as hypoxic vasoconstriction is one of the main mechanisms to avoid worsening arterial oxygenation. These vasodilator drugs can worsen hypoxemia by inhibiting this mechanism. Thus, appropriately designed trials are necessary to study the effect of these drugs on gas exchange in these patients.

## 7. Conclusions

The growing number of published papers about CPFE demonstrates the increasing interest in this phenotype, which encompasses a particular clinical, functional, and radiological profile. Associated PH is a common complication that it is important to identify, as it is the principal determinant of reduced survival in these patients. The overlapping of a wide variety of radiological and histopathologic lesions described in this syndrome supports the hypothesis that smoking habit as the main etiologic factor involved can generate diverse lung parenchymal disorders with different phenotypic expressions. Better understanding of its physiopathology and the molecular mechanisms involved will make it possible to develop future therapeutic strategies. It should not be forgotten that the prevention and treatment of smoking would possibly have the major impact on the natural history of this entity.
